# BDNF Val66Met polymorphism and protein levels in Amniotic Fluid

**DOI:** 10.1186/1471-2202-11-16

**Published:** 2010-02-08

**Authors:** Annamaria Cattaneo, Luisella Bocchio-Chiavetto, Roberta Zanardini, Eleonora Marchina, Daniela Bellotti, Elena Milanesi, Stefania Moraschi, Francesca Calabrese, Sergio Barlati, Marco Andrea Riva, Massimo Gennarelli

**Affiliations:** 1 Genetics Unit, IRCCS San Giovanni di Dio, Fatebenefratelli, Brescia, Italy; 2 Division of Biology and Genetics, Department of Biomedical Sciences and Biotechnology, University of Brescia, Italy; 3 Neuropsychopharmacology Unit, IRCCS San Giovanni di Dio, Fatebenefratelli, Brescia, Italy; 4 Center of Neuropharmacology, Department of Pharmacological Sciences and Center of Excellence on Neurodegenerative Diseases, University of Milan, Italy

## Abstract

**Background:**

Brain-Derived Neurotrophic Factor (BDNF) is a neurotrophin which plays survival- and growth-promoting activity in neuronal cells and it is involved in cellular plasticity mechanisms as it controls activity dependent synaptic transmission. A functional polymorphism (Val66Met) in the pro-region of BDNF, which affects the intracellular trafficking of proBDNF has been associated with memory and cognitive deficits as well as to an increased susceptibility for several psychiatric disorders especially those with a neurodevelopmental origin. To date, no study has evaluated the influence of the Val66Met polymorphism on BDNF levels in a peripheral system that may reflect fetal neurodevelopment. Therefore we investigated in amniotic fluids (AF) obtained from 139 healthy women during 15-17 week of pregnancy, BDNF protein levels in correlation with the Val66Met polymorphism.

**Results:**

Interestingly we found a significant BDNF protein levels reduction in 55 Met carriers (Val/Met and Met/Met) (p = 0.002) as compared to 84 non carriers (Val/Val), and no effect of fetus gender, maternal age or gestation week on BDNF levels has been observed.

**Conclusion:**

These results, although explorative, indicate that during fetal life the Val66Met genotype might influences BDNF protein levels in AF supporting the involvement of this polymorphism in behavioral and functional brain individual differences in the adulthood.

## Background

Brain-Derived Neurotrophic Factor (BDNF) is a neurotrophin widely expressed in the brain, which regulates neuronal survival, growth and connectivity during development and participates in synaptic plasticity mechanisms throughout adult life [1]. BDNF has been detected in several brain regions with the highest expression levels in hippocampus, cortex and cerebellum and in different non neuronal tissues like thymus, liver, spleen, heart, lung, immune system cells and Amniotic Fluid (AF) [2,3]. Interestingly, altered BDNF AF levels were found associated with Central Nervous System (CNS) abnormalities of the fetus, suggesting that AF BDNF levels could be indicative of fetal CNS development [4].

BDNF protein is first synthesized as a glycosylated precursor (proBDNF) that is then converted intracellulary to mature BDNF protein, the latter being stored and released upon cell stimulation [5]. An aminoacid substitution from Valine to Methionine due to a single-nucleotide polymorphism at the codon 66 has been identified in the propeptide region of BDNF (Val66Met). Egan and co-workers have demonstrated "in vitro" the functional relevance of this substitution since the genotype can affect the intracellular trafficking of proBDNF leading to reduced activity-dependent secretion of the neurotrophin. In turn, lower activity-dependent secretion of the neurotrophin is associated with the Met allele as compared to the Val allele [6,7]. The functionality of the polymorphism has also been supported by several findings in humans that reported an effect of genotype on memory and cognitive performances [6,8-10]. Moreover, recent magnetic resonance imaging (MRI) studies evidenced an effect of the Val66Met polymorphism on brain morphology, with Met carriers having reduced volume of different brain structures, including hippocampus, parahippocampal gyrus and prefrontal cortex [11,12].

Furthermore, the Val66Met polymorphism has also been widely investigated as a genetic susceptibility risk factor for a large spectrum of neuropsychiatric disorders, in particular those with a neurodevelopmental origin [13-17] leading often to contrasting results. Conversely, positive associations of the BDNF Val66Met have been reported with specific symptoms of depressive disorders [18], or schizophrenia and again, with specific illness morpho-functional endophenotypes [19] associated to brain development and functionality like a reduced hippocampal volume or a deficit in cognitive performances [19-21].

To date, at our best knowledge, no study has evaluated the putative influence of the Val66Met polymorphism on BDNF levels during pregnancy, a period during which fetal brain development appears vulnerable to external stimuli. In fact, it is well accepted that adverse events occurring during fetal life have profound and persistent effects on brain functions and may represent risk factors for psychopathologies later in life [22,23].

Therefore the aim of this study was to analyze putative functional effects of the BDNF Val66Met polymorphism in the fetal environment investigating correlation between BDNF protein levels and genotype in a sample of AFs obtained at the 15th-17th gestational week of physiological pregnancies.

## Results

The BDNF fetal genotype distribution in our study population was 3.6% (5/139) for Met/Met, 36% (50/139) for Val/Met and 60.4% (84/139) for Val/Val. Genotype distribution had no deviation from Hardy-Weinberg equilibrium (χ^2 ^= 0.55, p = 0.46, HWE program, John Ott version 1.10). Since all the studies that evaluated the functionality of this polymorphism assembled Val/Met individuals with Met/Met and it has also been demonstrated that the Met allele has a dominant effect, we performed our analyses subdividing our study-sample into carriers (Met66Met, Val66Met) and non carriers (Val66Val homozygotes) of the Met allele.

We firstly assessed whether maternal age or gestational week was significantly different in Met carriers versus non carriers, but we didn't find any difference (mean values ± SD: gestational age = 16.2 ± 2.4 in Val/Val and 15.9 ± 0.7 in Met carriers, p = 0.314; maternal age = 35.4 ± 3.7 in Val/Val and 36.2 ± 3.1 in Met carriers); there was also a similar fetal gender distribution among carriers and non carriers of the Met allele (49.1% of females in Met carriers and 44.0% of females in non carriers).

**Figure 1:**
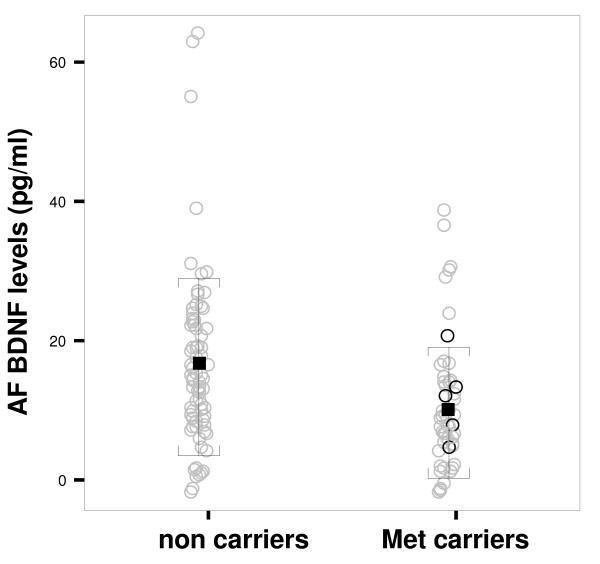
**BDNF protein levels distribution in 139 AFs (84 Val66Val individuals and 55 Met66 carriers)**. BDNF protein levels, evaluated by ELISA method, are significantly reduced in Met carriers versus non carriers The amount of BDNF expressed in pg/ml was 10.02 ± 9.62 for Met allele carriers and 15.88 ± 12.71 for non carriers (Mann-Whithney test: p = 0.002). In the group of Met allele carriers, the Met66Met are evidenced in black. Error Bars indicates the mean of values ± standard deviation.

We subsequently evaluated BDNF levels in AFs and we found that BDNF protein levels were significantly lower in Met allele carriers when compared to non carriers. In fact, as shown in Figure 1, the amount of BDNF expressed in pg/ml was 10.02 ± 9.62 for Met carriers and 15.88 ± 12.71 for non carriers (Mann-Whithney Test: p = 0.002). In addition no effect of the covariates maternal age, gestational week and fetal gender was found on BDNF levels (p = 0.672, p = 0.833 and p = 0.194 respectively).

## Discussion

This study represents the first evidence of an effect of the BDNF Val66Met polymorphism on protein levels during the 15-17 weeks of pregnancy.

Egan and colleagues [6] have shown in their seminal paper the functional relevance of the Val66Met polymorphism, demonstrating that BDNF genotype affects the intracellular trafficking of the neurotrophin, suggesting that there may be a specific 'trafficking' signal in the pro-domain of BDNF, which is required for an efficient sorting of the neurotrophin to the regulated secretory pathway. Moreover, they demonstrated that the genotype can also affect the activity-dependent release of BDNF [5] as recently confirmed by Chen and colleagues [24]. The decreased intracellular BDNF trafficking and release due to the presence of the Met allele are thought to underlie several dysfunctions in humans. In fact, individuals carrying the Met allele show reduced grey matter volumes within brain regions known to participate in verbal memory and visuospatial abilities [25] as well as altered hippocampal volume and gray matter density in the frontal and prefrontal cortex [11,12]. Furthermore, Stern and colleagues [26] have recently demonstrated a brain deficiency in N-acetyl-aspartate levels, an indicator of neuronal integrity, in subjects carrying the Met allele, suggesting that this allele could play an important role on hippocampal functionality and vitality and may have putative implications for several psychiatric disorders. To this support, a marked atrophy in several limbic regions, was reported in patients with Major Depression, Schizophrenia, Bipolar Disorder and other psychiatric disorders [27], suggesting the presence of a deficiency in neurogenesis and in synaptic plasticity, mechanisms highly regulated by BDNF. Moreover, the Val66Met polymorphism was also associated with alterations in amygdala volume and in its activity in response to emotional stimuli, suggesting an association between the Met allele and an increased vulnerability for anxiety disorders [28]. This polymorphism has been extensively studied in relation to several psychiatric disorders reporting often conflicting results. In particular, both alleles have been associated with different mental disorders and to date it is unknown how the two variants may exert their influence on disease susceptibility. Probably, a mental disorders or a cognitive or brain imaging endophenotype may be associated with the presence of the Met allele, while a distinct condition may have been associated with the Val variant. At this regard a recent case-control study in psychiatric disorders [29] reported that the Met allele significantly increases the risk for schizophrenia and eating disorders whereas exerts a protective effect for substance-related disorders.

Furthermore, studies analyzing gene-environment interactions [30,31] reported that BDNF Met carriers exposed to early life stress events (ELS) have smaller hippocampal and amygdala volumes, heart rate elevations, a decline in working memory and higher depression symptoms. In contrast, the combination of the BDNF Val/Val genotype and ELS is associated with increases in amygdala and prefrontal cortex grey matter and with higher anxiety symptoms.

It is well known that human brain undergoes complex morphological and functional changes during fetal development. In particular, during the first and second trimester of pregnancy, cortical subplate reaches its peak of development, and cortical neurogenesis, characterized by proliferation, migration and organization of neuronal connections, is predominantly taking place [22,23]. Therefore, during this period the immature brain may be particularly sensitive and vulnerable to a range of intrauterine influences like stress exposure and inflammation. During the first weeks of pregnancy Amniotic Fluid derives principally from maternal plasma, getting through to fetal membranes. Later, from tenth to twentieth week, bidirectional diffusion between placenta, umbilical cord and fetal skin occurs and in this period the AF composition becomes very similar to fetal plasma. Therefore, it may be assumed that the AF BDNF content in this period reflects the total circulating neurotrophin content of fetus [32].

In parallel, circulating BDNF levels in fetus could reflect those in the brain since a correlation between blood BDNF levels and the cortical protein expression has been observed during neurodevelopment in rats [33]. In this context, alterations of AF BDNF levels induced by the presence of the BDNF polymorphism in a critical period for brain development (15-17 weeks of pregnancy), in concomitance with other susceptibility genes or adverse intrauterine events, may represent a vulnerability risk factor for brain development and maturation. As previously reported, BDNF plays an important role in neural proliferation, survival and differentiation; therefore, reduced BDNF levels during CNS development could influence the correct morphology of specific cerebral regions as well as the cognitive and memory abilities that are widely reported to be affected in healthy subjects carrying Met allele. It is interesting to notice that in the adulthood the Val66Met polymorphism does not affect the levels of the neurotrophin in serum and plasma [34,35] and to date only Lang and colleagues [36] reported a significant serum BDNF increase in healthy subjects carrying Met allele. However, in this study the authors did not take in account the influence of additional factors, such as drugs, life style, environment and stress-life events, which interfere with BDNF levels. Moreover, another possible explanation for the discrepancy with our results could be the specific impact of the Val66Met polymorphism on BDNF levels during brain development and not in adult life, where this effect could be hidden by confounding factors putatively associated with the life style.

Some limitations of our study have to be mentioned. The data about AF BDNF levels are from one point in time, and the relationship between BDNF genotype and AF BDNF levels may be different at other times during gestation. Additionally, this study is correlational in nature and we cannot draw any definitive statements about the consequences of BDNF Val66Met genotype on AF protein levels. Larger studies in wider and better characterized samples are needed to confirm these findings.

## Conclusions

In conclusion, this study represents the first evidence of the functionality of the BDNF Val66Met polymorphism in the fetal environment showing a correlation between the genotype and BDNF protein levels in AF during neurodevelopment. Although these results are explorative, they support a role of this polymorphism in the individual differences in brain morphology and function, and in mental disorder susceptibility.

## Methods

### Sample collection

All the 139 amniotic fluid samples used in the study were obtained by amniocentesis performed between the 15 and 17 weeks of pregnancy (mean of gestational week: 16.1 ± 1.9) for fetal karyotype analysis requested by mothers for their advanced age (mean of maternal age = 35.7 ± 3.5). The inclusion/exclusion criteria were: healthy women with Caucasoid origin, fetuses with 46 XX or 46 XY karyotypes, no infections or complications during the previous gestational weeks and during the amniocentesis. The fetus gender was so distributed: 73 males and 61 females.

After amniotic fluid collection, each sample was centrifuged: the supernatant (AF) not necessary for alpha fetoprotein dosage was taken anonymously and stored at -80°C. Pellet fractions (AF cells, AFc) were cultured firstly in Amniochrome medium, which was replaced after 6-7 days with RPMI supplemented with 2 mM L-glutamine and 20% of Fetal Bovine Serum (FBS) as for routinely karyotype analysis. The leftover of AFc not necessary for karyotype analysis was collected anonymously without name or other detail, but with a number and bar-code. The study protocol has been approved by the local ethic committee (CEIOC).

### DNA purification and genotyping

DNA was isolated from AF cells using TRIZOL reagent, according to the manufacturer' protocol (Invitrogen) and was quantified by Spectrophotometer (NanoDrop Technologies, Delaware, USA). Primer pairs used to amplify the sequences containing Val66Met were: BDNF forward primer: AGGTGAGAAGAGTGATGACC and BDNF reverse primer: CTGGACGTGTACAAGTCTGC.

The PCR reactions were performed in a final volume of 25 μl with 2 μl of genomic DNA (100 ng/μl), 0.1 μl of each primers pairs (100 μmol/ul), 0.7 mM of each dNTPs (Eppendorf), 0.5 μl of MgCl2 2 mM, 1.25 units of Taq Polymerase (Bioline) and buffer supplied by the manufacturer (Bioline). The PCR products were then sequenced using an ABI BigDye Terminator Cycle Sequencing Kit and by 3130 XL DNA Sequencer Analyzer (Applied Biosystem).

### BDNF determination in Amniotic Fluids

BDNF levels in AF samples (n = 139) were measured by ELISA method using the human BDNF Quantikine kit (R&D Systems, Minneapolis, USA). The BDNF content was expressed as equivalent of human recombinant BDNF protein (pg of BDNF protein/ml of AF). The minimum detectable dose of the assay is typically less than 20 pg/ml.

### Statistical analysis

Analysis was performed using Statistical Product and Service Solutions 13.0 Version (SPSS). Results are given as mean value ± standard deviation. The Mann-Whithney test was used to evaluate differences in quantitative variables. The Pearson coefficient was used to evaluate bivariate correlations.

## Authors' contributions

**AC **Study design, data analyses and manuscript writing; **LB-C **Study design, data analyses and manuscript writing; **RZ, SM, **biochemical analyses; **FC, EM **genotyping analyses; **EM, DB **sample collection and clinical characterization; **SB**, **MAR **and **MG **Study design and manuscript critical revision. All the authors have contributed and approved the final manuscript.
